# TRAPPC9 Mediates the Interaction between p150^Glued^ and COPII Vesicles at the Target Membrane

**DOI:** 10.1371/journal.pone.0029995

**Published:** 2012-01-18

**Authors:** Min Zong, Ayano Satoh, Mei Kuen Yu, Ka Yu Siu, Wing Yan Ng, Hsiao Chang Chan, Julian A. Tanner, Sidney Yu

**Affiliations:** 1 School of Biomedical Sciences, The Chinese University of Hong Kong, Shatin, N.T., Hong Kong SAR, People′s Republic of China; 2 The Research Core for Interdisciplinary Science, Okayama University, Okayama, Japan; 3 Epithelial Cell Biology Research Center, The Chinese University of Hong Kong, Shatin, N.T., Hong Kong SAR, People′s Republic of China; 4 Department of Biochemistry, The University of Hong Kong, Pokfulam, Hong Kong SAR, People′s Republic of China; Institute of Molecular and Cell Biology, Singapore

## Abstract

**Background:**

The transport of endoplasmic reticulum (ER)-derived COPII vesicles toward the ER-Golgi intermediate compartment (ERGIC) requires cytoplasmic dynein and is dependent on microtubules. p150^Glued^, a subunit of dynactin, has been implicated in the transport of COPII vesicles via its interaction with COPII coat components Sec23 and Sec24. However, whether and how COPII vesicle tether, TRAPP (Transport protein particle), plays a role in the interaction between COPII vesicles and microtubules is currently unknown.

**Principle Findings:**

We address the functional relationship between COPII tether TRAPP and dynactin. Overexpressed TRAPP subunits interfered with microtubule architecture by competing p150^Glued^ away from the MTOC. TRAPP subunit TRAPPC9 bound directly to p150^Glued^ via the same carboxyl terminal domain of p150^Glued^ that binds Sec23 and Sec24. TRAPPC9 also inhibited the interaction between p150^Glued^ and Sec23/Sec24 both in vitro and in vivo, suggesting that TRAPPC9 serves to uncouple p150^Glued^ from the COPII coat, and to relay the vesicle-dynactin interaction at the target membrane.

**Conclusions:**

These findings provide a new perspective on the function of TRAPP as an adaptor between the ERGIC membrane and dynactin. By preserving the connection between dynactin and the tethered and/or fused vesicles, TRAPP allows nascent ERGIC to continue the movement along the microtubules as they mature into the cis-Golgi.

## Introduction

Vesicles budding from the donor membrane compartment need to translocate to the target compartment in order to achieve a net transfer of cargo proteins from one location to another. In the early secretory pathway, the involvement of microtubules in vesicular transport processes was evident more than two decades ago, as cells treated with the microtubule depolymerizing agent nocodazole had fragmented Golgi [1], [2], [3]. Manipulating the expression of certain motor proteins or subunits of the dynactin complex also disrupt the integrity of the Golgi and other membrane compartments in the early secretory pathway [4], [5], [6], [7], [8]. The ER exit sites are relatively immobile, so COPII vesicles are thought to be limited to short-range movement, although long-range movement has also been observed [9], [10]. It has been demonstrated that COPII vesicles are found at the growing ends of microtubule tracks, indicating that the COPII vesicle-microtubule interaction is carried out by a “search and capture” mechanism, although subsequent studies demonstrated that this mechanism may not be necessary for COPII vesicle transport [7], [11]. Nonetheless, dynein/dynactin is required for the movement of vesicles from ER to Golgi [12]. The precise mechanism of how p150^Glued^, a subunit of dynactin, directs COPII vesicle transport remains largely unknown. p150^Glued^ binds to coat subunits Sec23 and Sec24 at its carboxyl terminal cargo binding domain, called CT^Glued^ [11]. In cells overexpressing CT^Glued^, vesicular transport at the step of ER to Golgi is reduced, presumably by competitively inhibiting the interaction between endogenous p150^Glued^ and Sec23/Sec24 at the ER exit sites. Therefore, p150^Glued^ couples COPII vesicles onto microtubule tracks to allow the movement of COPII vesicles. p150^Glued^ is known to stimulate the motor processivity of cytoplasmic dynein [13], thereby allowing efficient movement of COPII vesicles once they are associated with microtubules.

p150^Glued^ has been implicated in a number of cellular processes including the integrity of the Golgi, as depletion of p150^Glued^ by siRNA results in fragmentation of the Golgi [7], [8]. It was thought that p150^Glued^ depletion causes disruption of microtubule integrity, which is required for Golgi integrity. The depletion of p150^Glued^ can cause morphological changes in ER exit sites with a more diffused staining pattern but does not slow down the rate of ER to Golgi transport [7]. This particular observation was not entirely conclusive as the degree of siRNA depletion of p150^Glued^ was roughly 50%. A more efficient p150^Glued^ depletion may be required to affect the rate of ER to Golgi transport. We have also observed that in cells depleted with p150^Glued^, ER exit sites, shown by the marker Sec31A, were dispersed from the perinuclear region to small puncta (Figure S1). COPI staining, which is enriched in the ERGIC and cis-Golgi, is also dispersed. These results suggest that the function of p150^Glued^ on the ER exit sites and/or COPII vesicle cannot be overlooked but do not provide a clear description for the role of p150^Glued^ on COPII vesicle transport.

COPII vesicles are tethered by a protein complex called TRAPP (Transport protein particle) [14], [15]. Vesicle tethering must occur in order to facilitate subsequent docking and fusion with the target membrane. It has been postulated that TRAPP recognizes COPII vesicles at the target membrane compartments, the ERGIC or cis-Golgi, although this interaction may be initiated at the ER exit sites. TRAPP subunit TRAPPC3 (a.k.a. mBet3) is enriched in the ER exit sites and can interact with Sec23, providing the initial interaction between COPII coat and its tether [16], [17].

At least three forms of TRAPP complex with different subunit composition have been proposed based on biochemical and genetic studies, and emerging evidence suggests TRAPP complexes could be functionally and structurally more complicated than initially thought [18], [19], [20]. In particular, the distinction between different forms of TRAPP in mammalian system is less clear [18]. Thus far, it has been firmly established that TRAPP functions as a COPII vesicle tethering factor and has intrinsic guanine nucleotide exchange activity toward the small GTPase Ypt1/Rab1 [14], [16], [21], [22], [23].

The intimate relationship between p150^Glued^ and COPII vesicles is also manifested by their extensive colocalization. We have observed that approximately 70% of the transfected GFP-Sec24 signals colocalized with endogenous p150^Glued^ signals in COS cells (data not shown). The interaction between p150^Glued^ and Sec23/Sec24 establishes a coupling between vesicle budding and vesicle movement. The validity of this model relies on whether Sec23/Sec24 will be released from the vesicle once budding is completed. Recently, the question has been raised whether uncoating of COPII vesicle occurs immediately after vesicle budding [24]. The COPII coat can remain associated with the vesicle membrane after budding, and partial uncoating is sufficient to allow vesicle fusion to take place at the target membrane. In this scenario, Sec23 and Sec24 remain on the vesicle membrane and can serve as a platform for protein interactions, so that TRAPP and p150^Glued^ can continue to bind to Sec23 and/or Sec24 to mediate vesicle tethering and movement, respectively. Vesicle movement and tethering must also be coordinated so that the recognition between the vesicle and the target membrane and the subsequent formation of SNARE pairing can take place without interference from different vesicle movement speeds. Therefore, communication between vesicle movement, largely if not solely mediated by p150^Glued^, and vesicle tethering, mediated by TRAPP, must exist. The ERGIC membrane compartments also move along microtubules as they gradually mature into the cis-Golgi. As shedding of the COPII coat is an inevitable event after vesicle fusion, maintaining the movement of tethered and/or fused vesicles along the microtubule by p150^Glued^ has to rely on an interaction other than COPII coat proteins. The vesicle tether, TRAPP, is a prime candidate for such interaction. We investigated whether dynactin interacts with COPII tethering complex, TRAPP, and discovered a physical interaction between the p150^Glued^ and TRAPPC9. This interaction is mediated by the carboxyl terminal, cargo-binding domain of p150^Glued^ and is stronger than the p150^Glued^-Sec23/Sec24 interaction. These results suggest a new role of COPII tethering factor TRAPP as a protein complex that helps restrict COPII vesicles on the microtubules.

## Results

### TRAPPC9 is localized to the early secretory pathway

**Figure 1:**
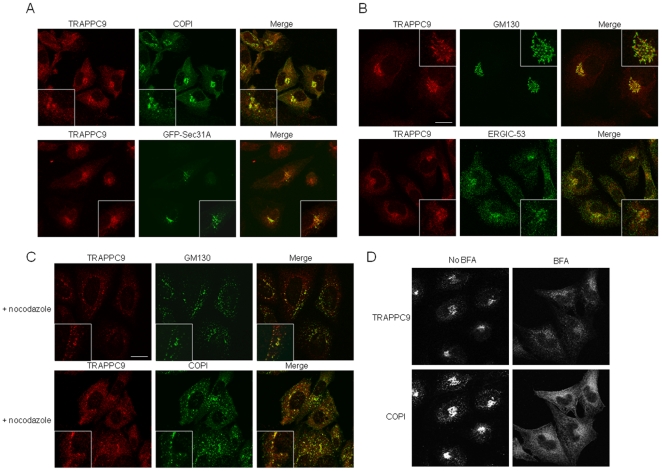
TRAPPC9 is predominantly enriched in cis-Golgi and ERGIC. CHO cells were stained with TRAPPC9 and various markers by indirect immunofluorescence. TRAPPC9 were detected by affinity purified rabbit IgG specific to TRAPPC9, followed by Alexa Fluor 568- conjugated goat anti rabbit secondary antibody. Organelle markers are detected by mouse monoclonal antibodies against the indicated proteins, followed by Alexa Fluor 488 conjugated goat anti- mouse secondary antibody. The extent of colocalization is presented in the merge images. The boxed areas are enlarged as insets. (A). TRAPPC9 (red) extensively colocalizes with COPI (green), which is enriched in the ERGIC and cis Golgi, and partially with transfected GFP-Sec31. (B). TRAPPC9 also extensively colocalizes with cis Golgi marker, GM130, and partially with ERGIC (ERGIC-53). (C). CHO cells treated with nocodazole for 1 h before fixed and stained with indicated antibodies. Small puncta derived from the dispersal of the Golgi membranes showed extensive overlap of TRAPPC9 signal (red) with GM130 (green, top panels) and COPI (green, bottom panels). (D). CHO cells treated with or without 10 µg/ml BFA for 1 hours before staining with antibodies against TRAPPC9 (top panels) or COPI (bottom panels). Scale bar = 20 µm.

In mammals, the TRAPP subunit TRAPPC3 is localized largely to ER exit sites [17], consistent with its unique function in binding to COPII coat protein Sec23. Other TRAPP subunits, including TRAPPC10, TRAPPC2 and TRAPPC4, are largely localized to the cis-Golgi [23]. TRAPPC9 has been reported to localize to the ER [25], but our data indicate that TRAPPC9 has limited localization at the ER exit sites (Figure 1A). More extensive colocalization was observed between TRAPPC9 and markers for ERGIC and cis-Golgi, including COPI (Figure 1A), ERGIC-53 and GM130 (Figure 1B). COPI vesicles bud from ERGIC and carry cargo proteins to the cis-Golgi [26], [27]. A more precise localization study using nocodazole to break up the membrane compartments into small punctate structures revealed that TRAPPC9 colocalized extensively with COPI and GM130 (Figure 1C). In cells treated with Brefeldin A (BFA), a majority of the TRAPPC9 signals became dispersed, suggesting these signals were associated with the ERGIC or cis-Golgi membranes (Figure 1D). These membrane structures are BFA-sensitive. Together, our results and those from others have established that TRAPP are predominantly localized to the ERGIC and the cis-Golgi, and to a limited degree, to the ER exit sites.

### TRAPP interferes with microtubule architecture

**Figure 2:**
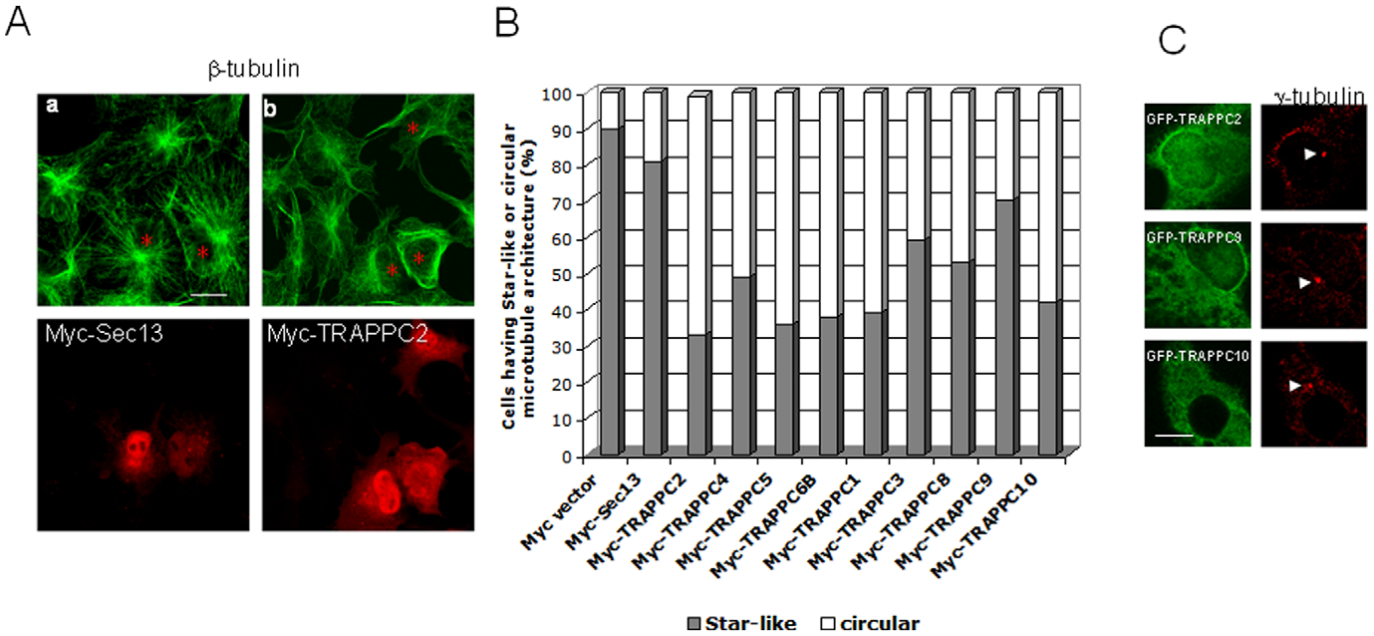
Overexpression of TRAPP subunits disrupts the microtubule “star-like” astral architecture in COS cells. (A) COS cells overexpressing the indicated Myc-tagged proteins are shown in the lower panels and the same cells are marked by red asterisks in the upper panels. Myc-tagged proteins were stained with Rhodamine-conjugated mouse anti-Myc antibody. Microtubules were stained with mouse antibody against β-tubulin, followed by Alexa Fluor 488 conjugated goat anti mouse secondary antibody. (B) Quantification of the status of microtubule architectures in COS cells overexpressing various TRAPP subunits. In all samples, at least 100 cells were counted. (C) TRAPP overexpression did not disrupt the MTOC. COS cells transfected with the indicated TRAPP subunits were stained with MTOC marker γ-tubulin (red). The integrity of the MTOC can be visualized by a single intense fluorescent dot (arrows). Scale bar = 20 µm (A);  = 10 µm (C).

Our initial evidence for the relationship between TRAPP and microtubules came from the observation that overexpression of various TRAPP subunits can rearrange microtubule arrays in COS cells. In normal COS cells, the microtubule tracks radiate from the MTOC with a star-like astral architecture (Figure 2Aa). Overexpression of various TRAPP subunits in COS cells can disrupt the architecture of microtubules in such a way that the astral appearance becomes disorganized, frequently presenting as a circular array. One such example is TRAPPC2 overexpression (Figure 2Ab, asterisks). This leads to Golgi membrane fragmentation much like the effect of nocodazole treatment (data not shown). We further quantified this array interfering effect induced by TRAPP subunits and found that virtually all TRAPP subunits tested have this effect at varying degree (Figure 2B). The varying degree of interference by different TRAPP subunits is likely a result of the difference in protein expression of these TRAPP subunits. Large subunits like TRAPPC8, TRAPPC9 and TRAPPC10 are generally expressed at a lower level than the smaller subunits, and therefore, had lower percentage of cells showing circular arrays (Figure 2B). GFP-tagged TRAPP subunits acted similarly (Figure S2), suggesting the effect is independent of the tag. The extent of interference is similar to that observed for overexpression of a subunit of the dynactin complex, p50 (Figure S2, second bar) [28]. This effect is specific as myc-tagged Sec13, a COPII coat subunit, showed little, if any, effect on microtubule arrangement (Figure 2Aa, asterisks). We confirmed that the MTOC remains intact in cells overexpressing various TRAPP subunits as indicated by the presence of an intense single focus of γ-tubulin signal juxtaposed by the nucleus (Figure 2C, arrowheads). These results indicate that an interaction between TRAPP and a microtubule-associated protein must exist. The overexpression of TRAPP subunits does not affect the MTOC, but must compete away the microtubule-associated protein from its native location on the microtubule. This upsets the proper interaction between TRAPP and the microtubule, resulting in abnormal microtubule arrangement.

### TRAPPC9 interacts with p150^Glued^

**Figure 3:**
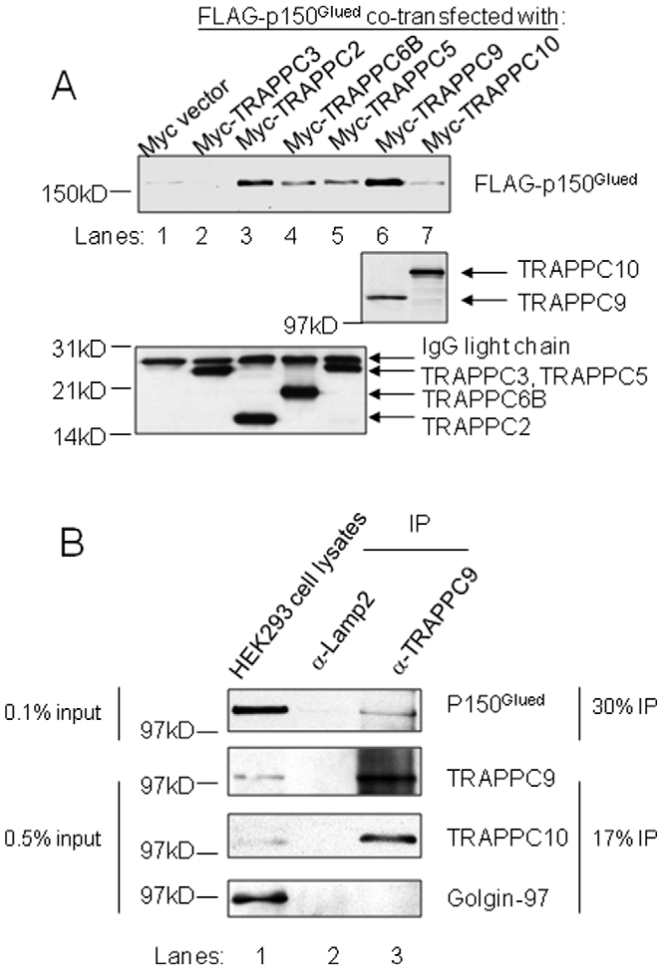
p150^Glued^ interacts with TRAPP. (A) The indicated myc-tagged TRAPP subunits were overexpressed with FLAG-tagged p150^Glued^ in COS cells. Co-immunoprecipitations were performed using antibody against c-Myc. The presence of FLAG-p150^Glued^ in the pull-down is detected by immunoblotting with antibody against FLAG (top panel). The extent of Myc-tagged TRAPP subunits precipitated was determined in immunoblotting using antibody against c-Myc (middle and bottom panels). Approximately 1% of the input lysates and 25% of the immunoprecipitants were loaded. (B) HEK293 cell lysates were subjected to immunoprecipitation using antibody specific to TRAPPC9 or to LAMP2 (negative control). The presence of TRAPPC9 (top panel), p150^Glued^ (second panel), and TRAPPC10 (third panel) in the immunoprecipitants were detected by immunoblotting using antibodies specific to these proteins. As negative control, Golgin-97 was not detected in the immunoprecipitants (bottom panel). For TRAPPC9, TRAPPC10 and Golgin-97, approximately 0.5% of the input lysates and 17% of the immunoprecipitants were loaded. For p150^Glued^, approximately 0.1% of the input lysates and 30% of the immunoprecipitants were loaded.

p150^Glued^ is known to interact with COPII coat proteins Sec23 and Sec24, and TRAPP subunit TRAPPC3 mediates the tethering of COPII vesicles by binding to Sec23. Therefore, we hypothesized that p150^Glued^ could be the microtubule-associated protein that is affected by TRAPP subunit overexpression. We tested whether p150^Glued^ interacts with TRAPP by a co-immunoprecipitation experiment. In COS cells overexpressing various Myc-tagged TRAPP subunits together with FLAG-tagged p150^Glued^, we immunoprecipitated the Myc-tagged TRAPP subunits and detected whether FLAG-p150^Glued^ is present in the pull-downs. TRAPPC2 and TRAPPC9 were observed to interact with p150^Glued^ most strongly (Figure 3A, lanes 3 and 6). To a lesser degree, other TRAPP subunits were also able to interact with p150^Glued^. However, the interaction between TRAPPC9 and p150^Glued^ is the most consistent in our experience. It is likely that the interaction between p150^Glued^ and other TRAPP subunits could be a result of indirect interaction via endogenous TRAPPC9 in the precipitation. To confirm that the TRAPP complex indeed interacts with p150^Glued^ in vivo, we determined whether endogenous p150^Glued^ is associated with endogenous TRAPP complex isolated from HEK293 lysate by immunoprecipitation using an antibody specific to TRAPPC9. As shown in Figure 3B, immunoprecipitant from anti-TRAPPC9 antibody (Figure 3B, lane3), but not control anti-LAMP2 antibody (Figure 3B, lane 2) was able to co-precipitate p150^Glued^ and TRAPPC10, another TRAPP subunit. As a control, peripheral Golgi protein Golgin-97 was not present in either immunoprecipitant (Figure 3B, bottom panel). Similar co-IP experiments using lysates from Caco-2 and CHO cells also detected interaction between p150^Glued^ and TRAPPC9 (data not shown). Together, we have confirmed that a physical interaction between TRAPP complex and p150^Glued^ exists in vivo and this interaction is most likely mediated by TRAPPC9.

**Figure 4:**
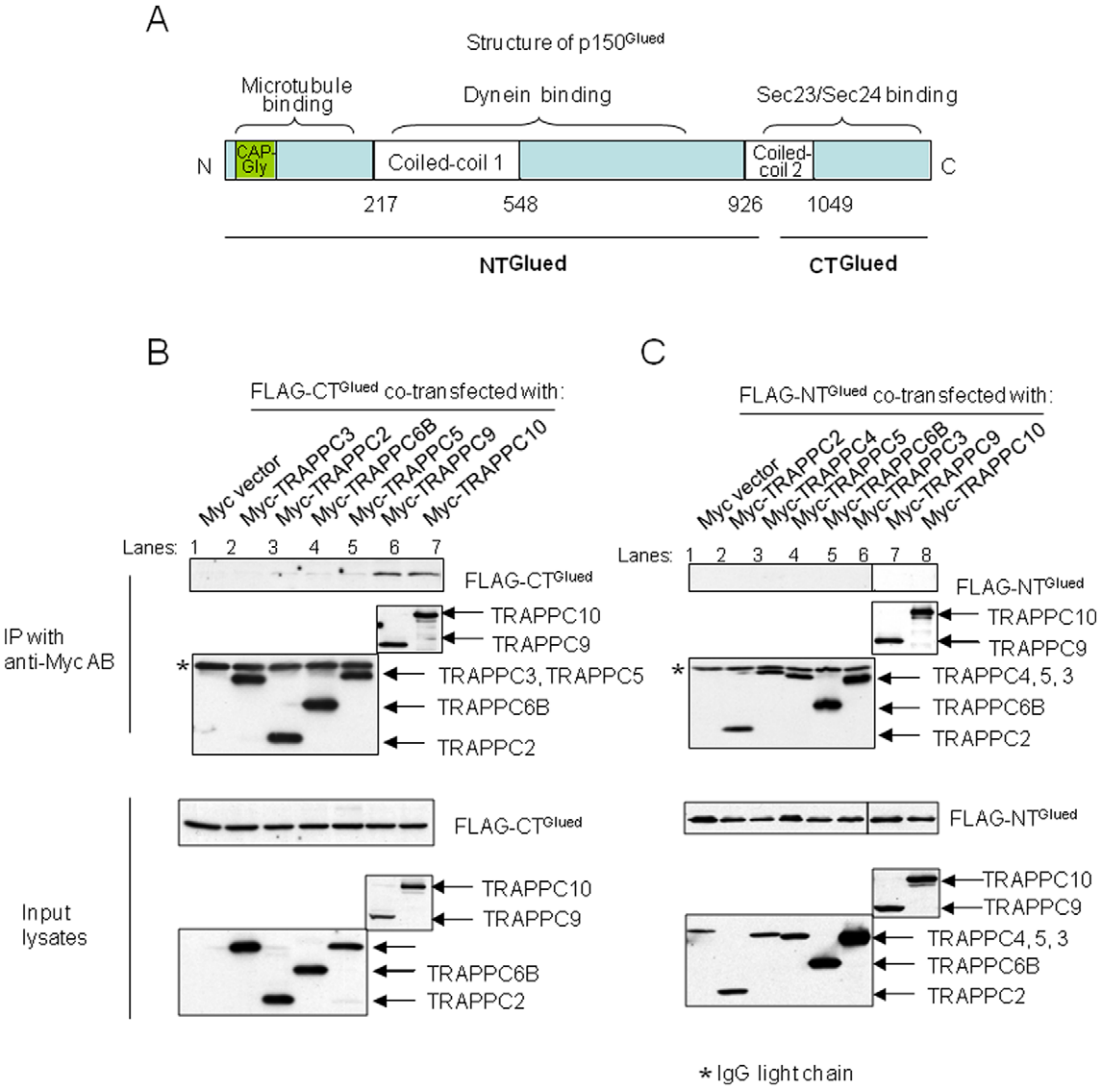
TRAPPC9 interacts with CT^Glued^. (A) Schematic diagram of p150^Glued^ structure. NT^Glued^ and CT^Glued^ were subjected to co-immunoprecipitation experiments. FLAG-tagged CT^Glued^ (B) and FLAG-tagged NT^Glued^ (C) were co-transfected with various Myc-tagged TRAPP subunits for immunoprecipitation. The extent of Myc-TRAPP subunit precipitation was determined by immunoblotting with anti-Myc antibody (middle panels). The presence of co-precipitated FLAG-CT^Glued^ or FLAG-NT^Glued^ was determined by anti-FLAG antibody (top panels). The level of protein expression in input lysates was determined by anti-FLAG or anti-Myc antibody (bottom panels). The precipitated anti-Myc IgG light chain was marked as asterisk in the immunoprecipitation samples. Approximately 1% of the input lysates and 25% of the immunoprecipitants were loaded.

We further investigated which domain of p150^Glued^ interacts with TRAPPC9. The interaction between Sec23/Sec24 and p150^Glued^ is mediated by the cargo-binding domain at the carboxyl terminus of p150^Glued^, called CT^Glued^ (Figure 4A). As shown in Figure 4B, CT^Glued^ binds to TRAPPC9 most strongly and consistently (lane 6). Weak and less consistent interactions were also detected with other TRAPP subunits, e.g. TRAPPC10 (Figure 4B, lane 7). The amino-terminal of p150^Glued^, NT^Glued^, did not bind to any TRAPP subunits tested (Figure 4C). Therefore, TRAPPC9 interacts with the cargo-binding domain of p150^Glued^, similar to Sec23 and Sec24.

**Figure 5:**
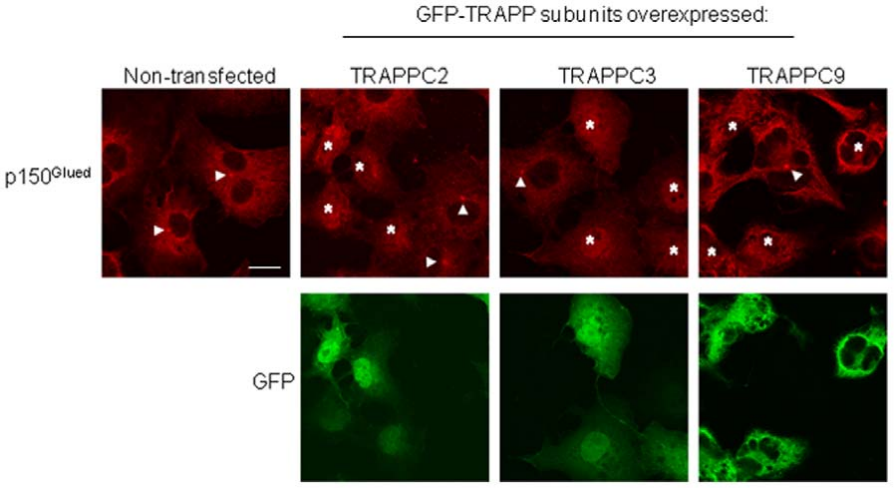
Overexpression of TRAPP subunits disrupts the MTOC localization of endogenous p150^Glued^. COS cells transfected with the indicated GFP tagged TRAPP subunits (bottom panels) were stained with endogenous p150^Glued^. The presence of p150^Glued^ signal at the MTOC can be observed in the non-transfected cells (Arrowheads). In cells transfected with the indicated TRAPP subunits, (asterisks, top panels), the signal of p150^Glued^ at the MTOC disappeared. Scale bar = 20 µm.

As overexpressing TRAPP subunits affects microtubule architecture without affecting the integrity of the MTOC, we hypothesized this effect could be due to a mislocalization of p150^Glued^ in TRAPP-overexpressing cells. In COS cells, p150^Glued^ is diffuse in the cytosol, at the microtubule plus ends at the cell periphery and at the MTOC (Figure 5, arrowheads). Overexpressing the indicated TRAPP subunits disrupted p150^Glued^ localization at the MTOC (Figure 5, asterisks), suggesting that the interfering effect on microtubule architecture by TRAPP overexpression is caused by mislocalization of endogenous p150^Glued^ in the TRAPP-transfected cells.

p150^Glued^ can be found in the cytosol and in the perinuclear region that includes the MTOC (Figure S3). When the cells were fixed with formaldehyde, a more diffuse cytosolic staining pattern with stronger signals around the perinuclear region was observed. Some of the p150^Glued^ perinuclear signals colocalize with TRAPPC9 (Figure S3A). These signals are different from the intense and dot-like structure found at the MTOC in methanol fixed cells (Figure 5). In cells treated with nocodazole, p150^Glued^ localization is dispersed into small punctate structures due to the absence of intact microtubules. Some of the p150^Glued^-positive punctate structures clearly colocalize with the TRAPPC9 puncta (Figure S3B). Higher magnification revealed that p150^Glued^ is closely associated with the TRAPPC9-positive puncta (Figure S3C, arrows). These results are consistent with a close association between p150^Glued^ and TRAPPC9 in vivo.

### TRAPPC9 competes with Sec23/Sec24 for p150^Glued^ binding

**Figure 6:**
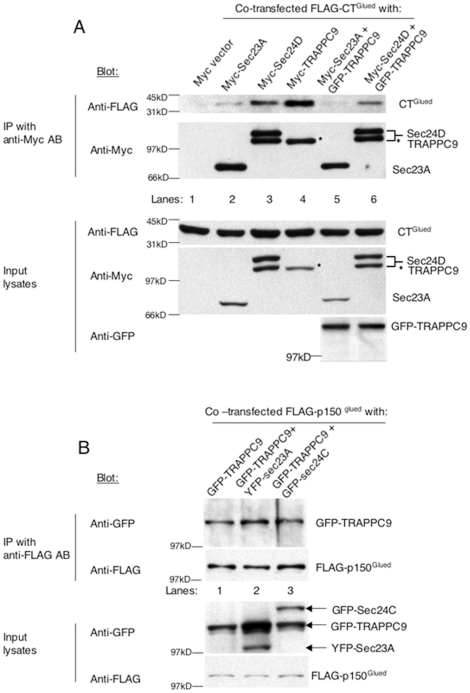
TRAPPC9 competes with Sec23 and Sec24 for binding to p150^Glued^. (A) Myc-Sec23 (lanes 2 and 5) or -Sec24 (lanes 3 and 6) were co-transfected FLAG-CT^Glued^ for co-immunoprecipitation experiments. The ability of TRAPPC9 to inhibit the interaction between Sec23 or Sec24 with CT^Glued^ was determined by the presence of GFP-TRAPPC9 (lanes 5–6). The amount of FLAG-CT^Glued^ co-precipitated was determined by immunoblotting with anti-FLAG antibody (top panel). The amount of Myc-tagged protein precipitated was determined by blotting with anti-Myc antibody (second panel). Levels of protein expression in the transfected cell lysates were determined by blotting the input lysates with the indicated antibodies (bottom three panels). (B) Transfected Sec23 or Sec24 lost the ability to bind to p150^Glued^ in the presence of co-transfected TRAPPC9. Transfected FLAG-p150^Glued^ was precipitated by antibody against FLAG (second panel). The presence of co-precipitated YFP-Sec23A, GFP-Sec24C or GFP-TRAPPC9 was detected by antibody against GFP (top panel). Only TRAPPC9, but not Sec23 or Sec24, could be co-precipitated with FLAG-p150^Glued^. The levels of protein expression in the transfected cell lysates were determined by blotting the input lysates with the indicated antibodies (bottom two panels). In both experiments shown in (A) and (B), approximately 1% of input lysates and 25% of the immunoprecipitants were loaded.

What is the functional relationship between TRAPPC9, p150^Glued^ and Sec23/Sec24? We performed co-immunoprecipitation to test if the presence of TRAPPC9 could interfere with the interaction between p150^Glued^ and Sec23/Sec24. In this experiment, Myc-tagged Sec23 or Sec24 was co-transfected with FLAG-tagged CT^Glued^. The effect of TRAPPC9 on p150^Glued^-Sec23/Sec24 interaction was assessed by co-transfection of GFP-TRAPPC9. The transfection conditions were carefully determined to achieve a comparable level of expression for each protein (Figure 6A, bottom three panels). The presence of TRAPPC9 strongly inhibited the abilities of Myc-Sec23 or Myc-Sec24 to pull-down FLAG-CT^Glued^ (Figure 6A, top panel, compare lanes 2 and 3 with lanes 5 and 6, respectively), suggesting that interactions between CT^Glued^ and Sec23 or Sec24 were inhibited by TRAPPC9. Furthermore, TRAPPC9 was better able to pull down CT^Glued^ than Sec23 or Sec24, suggesting a stronger interaction (Figure 6A, top panel, compare lanes 2 and 3 with lane 4). In a similar experiment, we immunoprecipitated FLAG-p150^Glued^ and determined how Sec23 and Sec24 compete with TRAPPC9 for p150^Glued^. As shown in Figure 6B, neither the co-transfected YFP-Sec23 nor GFP-Sec24C was able to compete with GFP-TRAPPC9 for the interaction of FLAG-p150^Glued^ (Figure 6B, top panel). Taken together, this experiment demonstrates that TRAPPC9 inhibits the interaction between p150^Glued^ and Sec23/Sec24, most likely by competitive binding to the cargo-binding domain, CT^Glued^.

**Figure 7:**
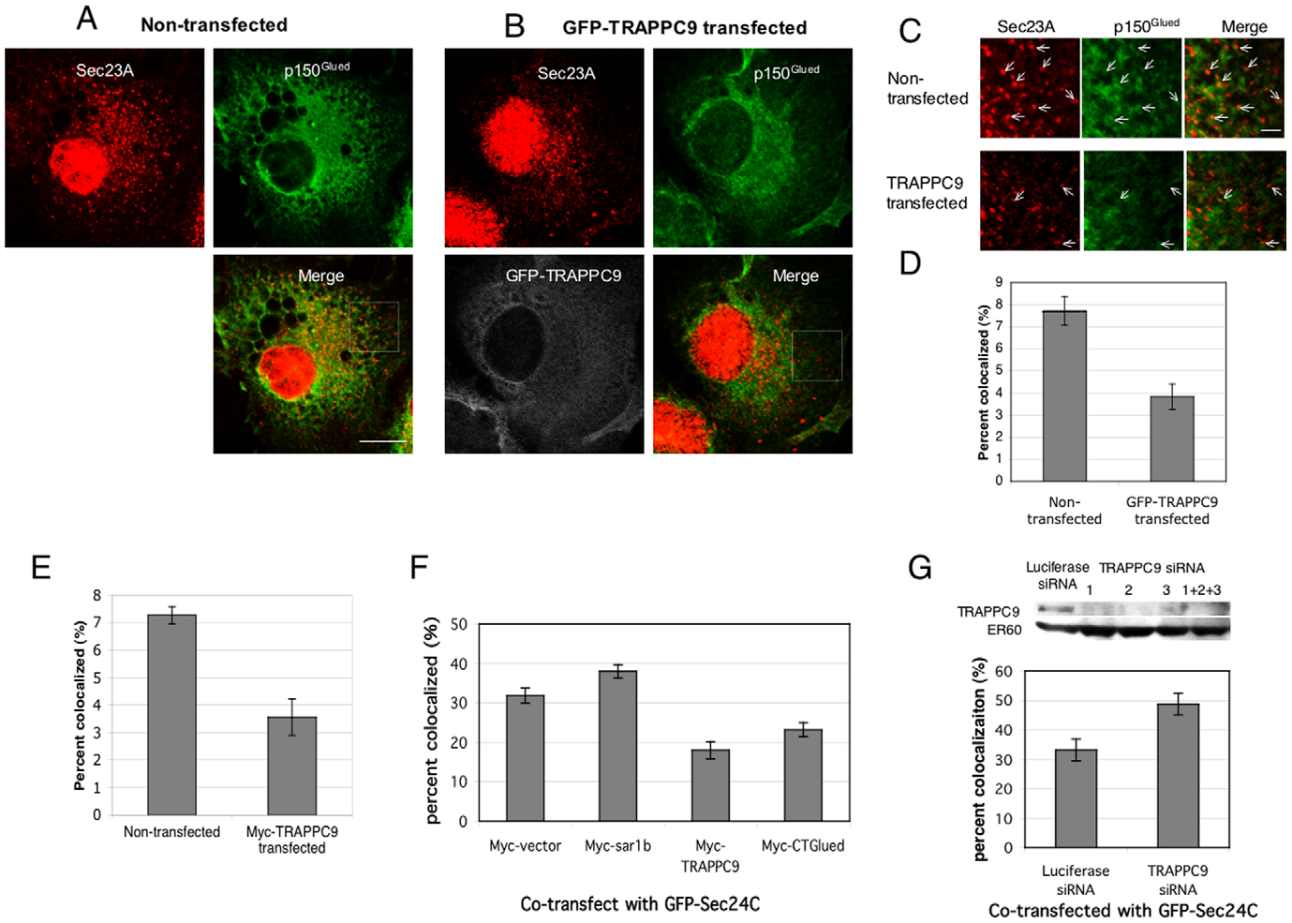
Overexpression of TRAPPC9 reduces the association of p150^Glued^ and ER exit sites in vivo. Non-transfected (A) or GFP-TRAPPC9-transfected (B) COS cells, were treated with nocodazole before staining with p150^Glued^ and Sec23A. p150^Glued^ signal was scanned by confocal microscopy at 633 nm and pseudo-colored in green. Sec23A was scanned at 543 nm and visualized in red; GFP-TRAPPC9 signal was scanned at 488 nm and visualized in white. Scale bar = 20 µm. The boxed areas are enlarged and presented in (C). Fluorescent dots of p150^Glued^ and Sec23A that are colocalized are indicated by arrows. Scale bar = 5 µm. (D) Quantifications of the colocalized signals in (C) as percentages of Sec23A-positive fluorescent dots that colocalized with p150^Glued^-positive dots. In non-transfected cells, 7.72% (S.E.M. = 0.648%) of the Sec23A dots were found to colocalize with p150^Glued^. A total of 890 Sec23A fluorescent dots in 8 cells were counted. In GFP-TRAPPC9 transfected cells, 3.83% (S.E.M. = 0.577%) Sec23A dots colocalized with p150^Glued^. 493 dots in 7 cells were analyzed. (E) Myc-TRAPPC9, instead of GFP-TRAPPC9, was transfected into COS cells. Sec23A was labeled with AF488-conjugated secondary antibody, p150^Glued^ was labeled with AF633-conjugated secondary antibody, and Myc-TRAPPC9 was labeled with TRITC-conjugated mouse anti-Myc antibody. In 11 non-transfected cells analyzed, a total of 1571 Sec23A-positive dots were analyzed and 7.27% (S.E.M. = 0.313%) colocalized with p150^Glued^-positive dots. In comparison, 3.56% (S.E.M. = 0.665%) colocalization was observed in Myc-TRAPPC9 transfected cells. 1087 Sec23A-positive dots in 10 transfected cells were analyzed. (F) Hela cells co-transfected with GFP-Sec24C and the indicated sequences in Myc-tagged vector. Endogenous p150^Glued^ was labeled with AF633-conjugated secondary antibody and the overexpressed Myc-tagged proteins were labeled with rhodamine-conjugated mouse anti-c-Myc IgG. Colocalized signals between p150^Glued^ and GFP-Sec24C were counted as above. Fluorescence signals from at least six cells were counted, with totally 262 dots or more for each sample. (G) HEK293 cells were transfected with Luciferase siRNA or three combined TRAPPC9 siRNAs for 72 hrs. Three TRAPPC9-specific siRNAs efficiently depleted the target protein as shown by immunoblotting (top panels). TRAPPC9 depletion significantly increased the co-localization of GFP-Sec24C and p150^Glued^ from 33.15% (S.E.M. = 0.369%) to 48.74% (S.E.M. = 0.22%). In this experiment, 473 fluorescent dots in 10 cells (control luciferase knockdown), and 494 dots in 11 cells (TRAPPC9 knockdown) were analyzed. Error bars = S.E.M.

If TRAPPC9 competes with Sec23/Sec24 for the binding with p150^Glued^, one will expect the overexpression of TRAPPC9 can reduce the association of p150^Glued^ and ER exit sites. We tested this hypothesis by overexpressing TRAPPC9 in COS cells and determined whether the extent of colocalization between p150^Glued^ and COPII coat components was reduced. In this experiment, we treated the transfected cells with nocodazole before fixation and staining, so that the signals of p150^Glued^ and Sec23 around the MTOC became dispersed, and the extent of colocalization can be quantified more easily by the number of colocalized fluorescence dots. COS cells were used because their flat morphology allows easy quantification of the fluorescence signals, despite the presence of non-specific nuclear signal labeled by the Sec23 antibody. The result indicated that TRAPPC9 disrupts the colocalization between ER exit sites and p150^Glued^. In cells overexpressing GFP-TRAPPC9 (Figure 7B–D), the extent of colocalization between p150^Glued^ and ER exit sites decreased from 7.72% non-transfected cells to 3.83% in GFP-TRAPPC9 transfected cells (Figure 7D). In a similar experiment, overexpressing Myc-TRAPPC9 also disrupted the colocalization between p150^Glued^ and ER exit sites. The extent of p150^Glued^ and ER exit sites colocalization decreased from 7.27% in non-transfected cells to 3.56% in Myc-TRAPPC9 transfected cells (Figure 7E). To further illustrate the effect of TRAPPC9 on disrupting the interaction between p150^Glued^ and ER exit sites, we co-transfected GFP-Sec24C and Myc-TRAPPC9 in Hela cells and determined if the colocalized signals between GFP-Sec24C and endogenous p150^Glued^ were reduced (Figure 7F). In cells co-transfected with GFP-Sec24C and Myc-vector, approximately 32% of the GFP-Seec24C fluorescence dots were colocalized with endogenous p150^Glued^ signals, as compared to approximately 7% of endogenous Sec23A signals colocalized with endogenous p150^Glued^ shown in Figure 7D. The reason for this increase is currently not clear but we have excluded the possibility that GFP-Sec24C overexpression could drive its interaction with p150^Glued^ because we observed a similar increase of colocalized signals between p150^Glued^ and ER exit sites by overexpressing GFP-Sec31A (data not shown). Co-transfection with Myc-TRAPPC9 decreased the p150^Glued^-GFP-Sec24C colocalized signals from 32% to 18%. As a positive control, we co-transfected Myc-CT^Glued^ with GFP-Sec24C. CT^Glued^ interacts with Sec23/Sec24 and TRAPPC9. Overexpression of CT^Glued^ disrupted the interaction between p150^Glued^ and ER exit sites and reduced the p150^Glued^-GFP-Sec24C colocalized signals to 23%. Sar1b did not cause any change in microtubule architecture and has not been reported to affect dynactin function. Therefore, we co-transfected Myc-Sar1b as a negative control. Co-transfection with Myc-Sar1b did not reduce the colocalization between GFP-Sec24C and p150^Glued^ as demonstrated by Myc-TRAPPC9 and Myc-CT^Glued^. In fact, Sar1b caused a slight increase in colocalization between GFP-Sec24C and p150^Glued^. We do not know the precise cause for this so far. Nonetheless, the result in Figure 7F is in agreement with Figure 7D and 7E, confirming the inhibitory effect of TRAPPC9 on the interaction between p150^Glued^ and ER exit sites. If the overexpression of TRAPPC9 is able to reduce the colocalization between p150^Glued^ with ER exit sites, one will expect depleting TRAPPC9 can increase such colocalization. In HEK293 cells depleted with TRAPPC9 by siRNA, we observed the colocalized signals between p150^Glued^ and GFP-Sec24C increased from 33.15% to 48.74% (Figure 7G). Therefore, TRAPPC9 overexpression could inhibit the interaction between COPII vesicles and p150^Glued^ in vivo and its depletion could increase the association between p150^Glued^ and COPII vesicles.

## Discussion

A close relationship between TRAPP and microtubules is evident from the previous report that the localization of TRAPPC3 is resistant to the treatment of brefeldin A but is disrupted by nocodazole [17]. Furthermore, the restoration of TRAPPC3 localization after removal of nocodazole can be achieved even in the presence of brefeldin A (data not shown), suggesting that proper localization of TRAPPC3 is predominantly dependent on microtubule integrity rather than membrane compartment integrity in the early secretory pathway. The depletion of TRAPPC3, however, did not cause major changes to microtubule architecture [17]. These observations are consistent with the hypothesis that TRAPP does not actively affect microtubule and motor function directly but relays the movement of cargo via an exchange of interaction from COPII coat and dynactin to COPII tether and dynactin. However, ectopic overexpression of TRAPP can compete p150^Glued^ away from its native location, thus indirectly affecting the integrity of microtubule architecture.

**Figure 8:**
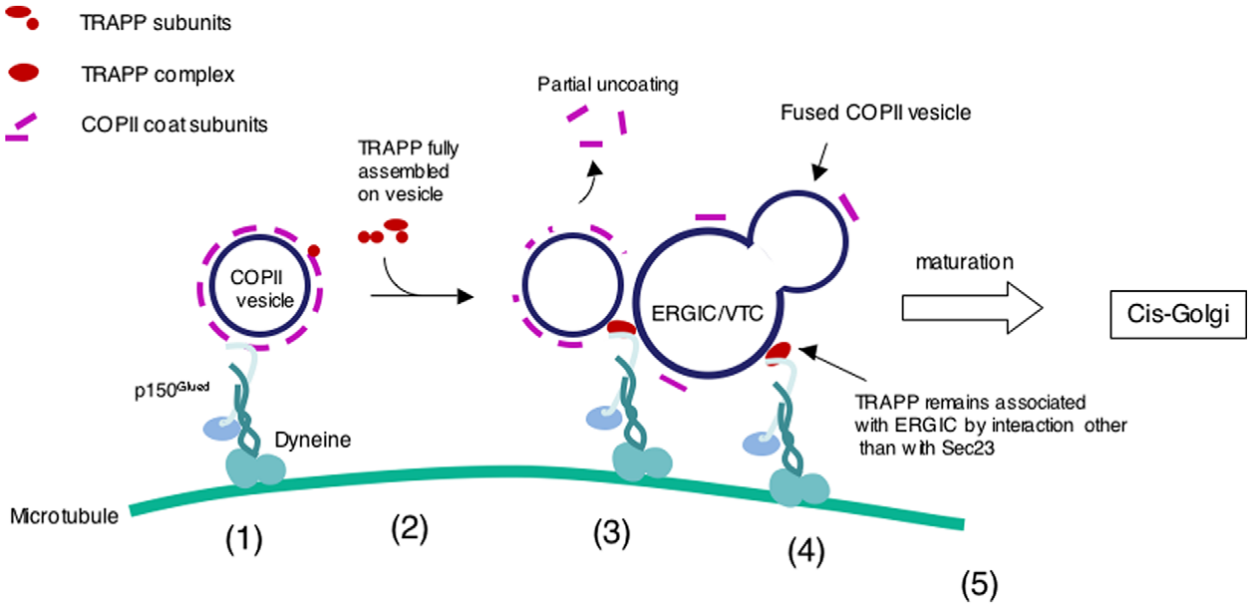
Schematic diagram depicting how TRAPP and p150^Glued^ coordinate the movement and tethering of a COPII vesicle is tethered at the ERGIC. (1). Dyneine-powered movement of a fully coated COPII vesicle along microtubule is mediated by an interaction between p150^Glued^ and Sec23/Sec24. (2). TRAPP subunits assemble onto the vesicle to form a fully functional TRAPP complex during the movement. (3). When the vesicle reaches the ERGIC, COPII coat will be partially shed to facilitate vesicle fusion but the interaction between p150^Glued^ and the vesicle is now mediated by the TRAPPC9-p150^Glued^ interaction. (4). TRAPP is anchored on the target membrane by a currently unknown mechanism. (5). The vesicle may fuse with the ERGIC but the TRAPPC9-p150^Glued^ interaction continues to help move the ERGIC towards the cis-Golgi.

Here, we started with the observation that TRAPP is functionally linked to the microtubules, and then identified that p150^Glued^, a subunit of dynactin, interacted with TRAPP via the subunit TRAPPC9. This novel interaction between p150^Glued^ and TRAPP adds a new perspective to the function of TRAPP. Vesicle tethering at the target membrane is viewed as the end point of a trafficking step. Here, our data suggest that tethering of COPII vesicles by TRAPP at the ERGIC may serve as a starting point for the movement of the tethered and/or fused vesicles, i.e., the nascent ERGIC, toward the cis-Golgi, with TRAPP now serving as an adaptor between the ERGIC and dynactin (Figure 8). In this model, when a COPII vesicle approaches the ERGIC (or another COPII vesicle in the case of homotypic vesicle tethering), a fully assembled TRAPP complex, which includes TRAPPC9, will activate Rab1 and serve as a tether for the COPII vesicle. At that point, TRAPPC9 will compete for the interaction of p150^Glued^ thereby shedding the Sec23/Sec24 dimer, thus relaying the interaction between vesicle and p150^Glued^, and at the same time, preparing the vesicle for docking and fusion. Preservation of the vesicle-dynactin interaction for the subsequent movement of ERGIC is a key feature of this model. It has been well documented that the ERGIC moves toward the cis-Golgi as the organelle gradually matures.

Thus far, it is not clear the extent to which COPII coat subunits are released from the budded COPII vesicle in vivo. It is possible that only partial uncoating is required for COPII vesicles to fuse with the target membrane [24]. In yeast, COPII vesicles remain coated until they reach the Golgi, where casein kinase I δ/Hrr25p phorsphorylates Sec23p to promote uncoating of the vesicles [29]. This finding suggests the uncoating of COPII vesicles at the target membrane happens concomitantly with other biochemical events such as vesicle tethering and fusion, and COPII coat components could be present at the target membrane. Indeed, it has previously been reported that Sec13-positive signals significantly overlap with ERGIC-53 [26], [30], suggesting remnant COPII coat can stay on the membrane even after COPII vesicle fusion. The remaining COPII coat may serve as a platform for protein interactions, permitting the binding of p150^Glued^ for vesicle movement and/or of TRAPP for tethering of other incoming vesicles. The TRAPPC9-p150^Glued^ may, in turns, indirectly promote shedding of Sec23 and Sec24 from the fused vesicles so that these membrane compartments will gradually assume the identity of the cis-Golgi.

The localization of TRAPPC9 at the target membrane, e.g. the ERGIC, will determine the precise timing of the dissociation of p150^Glued^ from Sec23/Sec24. Evidence suggesting TRAPPC9 is localized to the ERGIC also comes from cells transfected with a constitutively active form of Sar1, Sar1[H79G]. In Sar1[H79G] transfected cells, COPII coat proteins become concentrated around the MTOC due to impaired uncoating [31], [32]. Similar to the pattern of GFP-Sec24C, p150^Glued^ signal clustered at the MTOC in cells transfected with Sar1[H79G], suggesting that binding of p150^Glued^ to Sec23/Sec24 can occur before uncoating (Figure S4A, left panels). The TRAPPC9 signal becomes diffuse and cytosolic in this condition (Figure S4A, right panels). As a control, wildtype Sar1 did not cluster TRAPPC9 or GFP-Sec24C (Figure 4B). These data suggest that there is a fundamental difference in the way p150^Glued^ and TRAPPC9 interacts with COPII vesicles in that p150^Glued^ can bind to fully coated COPII vesicles but TRAPPC9 cannot “see” such fully coated vesicles to be clustered at the MTOC. This is different from the localization of TRAPPC3, which clusters at the MTOC like p150^Glued^ in Sar1[H79G] transfected cells (data not shown). Therefore it is likely that TRAPPC9 can only come into contact with COPII vesicle during uncoating, at which time it competes with Sec23/Sec24 for the interaction with p150^Glued^, and the only logical site for such event to take place is at the target membrane, the ERGIC. We speculate that the dissociation between Sec23 and Sec24 may be necessary to weaken the tripartite interaction sufficiently so that TRAPPC9 can successfully displace Sec23 and Sec24 for p150^Glued^. These results are consistent with our hypothesis that p150^Glued^ binds to COPII coat initially to conduct vesicle movement, and then TRAPP-mediated tethering of the vesicle at the target membrane will preserve the p150^Glued^-mediated movement of the tether and/or fused vesicles towards the cis-Golgi.

Two of the best-characterized biochemical functions of TRAPP are COPII vesicle binding via TRAPPC3 and Sec23 interaction, and GEF activity of Rab1/Ypt1p. In mammals, Sec23 is enriched in ER exit sites and Rab1 is enriched in ERGIC [33]. Paradoxically, both of these membrane compartments are different from the predominant localization of the TRAPP subunits at the cis-Golgi. We speculate here that the assembly of the TRAPP complex in vivo may be sequential, initiated by the binding of TRAPPC3 to Sec23 at the ER exit sites, and the complex is fully assembled at the ERGIC and remains on the membrane at steady state as the ERGIC matures into the cis-Golgi. The different behaviors of TRAPPC3 and TRAPPC9 with Sar1[H79G] overexpression seems to be in agreement with this notion. Indeed, sequential assembly of TRAPP complex via the conversion of TRAPPI to TRAPPII through the addition of the TRAPPII-specific subunits has been implicated in yeast [34]. Unlike TRAPPC3, the localization of TRAPPC9, as well as other TRAPP subunits, is sensitive to BFA, suggesting a dependence on Golgi membrane integrity. Therefore, even if TRAPP complex assembly is initiated on COPII vesicles, anchoring of TRAPP to the cis-Golgi must be mediated by yet another mechanism.

## Materials and Methods

### DNA and siRNA

cDNAs encoding mammalian TRAPP subunits were amplified by PCR and subcloned into pCMV-Myc (Clontech) or peGFP-Cx vector (Clontech). Myc-sec23A and Myc-sec24D were constructed by transferring the corresponding cDNA sequences into pCMV-Myc [16]. cDNA encoding human P150^Glued^ was purchased from Open Biosystem and transferred from vector pYx-Asc to pFLAG-CMV2 (Sigma). The carboxyl terminus of p150^Glued^ (amino acid 938–1254, CT^Glued^) was amplified from plasmid p150^Glued^ by PCR and subcloned into pFLAG-CMV2 and pCMV-Myc. siRNA targeting to human p150^Glued^: 5′-gacuucaccccuugauuaauu-3′ was designed according to Dixit et al [8]. siRNAs targeting to TRAPPC9 were designed using the algorithm designed by Invitrogen: The sequences of the oligoes are as follows: 1) 5′- gcugcugcguucugugaauuu; 2) 5′- gcauggaagcaucagaauuuu; 3) 5′- gcaacaaagcaggcgacuauu.

### Antibodies

Mouse monoclonal antibodies against c-Myc, 9E10 (sc-40), 9E10-conjugated with TRITC, and rabbit polyclonal antibody against GFP (sc-8334) were purchased from Santa Cruz Biotechnology, Inc. (CA, USA). Monoclonal antibody against Golgin-97, was a gift from Dr. Wing Keung Liu's laboratory (The Chinese University of Hong Kong), and was originally purchased from Invitrogen. Monoclonal antibody against Sec31A (612350), GM130 (610823) and p150^Glued^ (610474) were purchased from BD Biosciences. Monoclonal antibody against ERGIC-53 was obtained from Hans-Peter Hauri (University of Basel, Switzerland) [35]. Mouse monoclonal antibody against TRAPPC10, 5B4 (H00007109-M01) was purchased from Abnova corporation. Mouse monoclonal antibody against β-tubulin, TUB 2.1 (T4026), against γ-tubulin (T6557), against FLAG (Clone M2) was purchased from Sigma. Rabbit polyclonal antibody against LAMP2 was purchased from Alexis (210-206-R100). Alexa Fluor secondary antibodies were purchased from Invitrogen: 488 goat anti-mouse (A11017), 568 goat anti-mouse (A11019), 635 goat anti-mouse (31575), 488 goat anti-rabbit (A11070) and 568 goat anti-rabbit (A21069). Horseradish Peroxidase Conjugated secondary antibodies were purchased from Rockland: anti-mouse IgG (610–1302) and anti-rabbit IgG (211–1302). Rabbit polyclonal antibody against TRAPPC9 was raised to a GST fusion protein that contained amino acids 840–1143 of human TRAPPC9 [36].

### Cell culture and transfection

COS1, HEK293, Hela cells are maintained in Dulbecco's minimum essential medium (DMEM) (Invitrogen) supplemented with 10% fetal bovine serum (FBS) (Invitrogen). Chinese hamster ovary CHO-K1 cells were grown in DMEM supplemented 0.35 mM L-proline (Sigma), 10% FBS. All four cell lines were obtained from the ATCC (Manassas, VA). Transfection assay was performed with polyethylenimine (PEI) method (Godbey et al., 1999) or PolyJet (SL100688) according to the manufacturer's instructions (SignaGene Lab). 24–36 hours after transfection, the cells were harvested for analysis.

### Immunoprecipitation

Transfected COS1 cells in 10 cm tissue culture plates were lysed in lysis buffer (20 mM Tris, pH 8.0, 100 mM NaCl, 0.1% NP-40 and 1× protease inhibitor cocktail Complete® (Roche)). The cells were scraped down and collected in 1.5 ml tubes. After brief vortex, the lysates were centrifuged at 10,000 rpm at 4°C for 10 minutes. The supernatants were subject to immunoprecipitation with 1–2 µg of anti-Myc or anti-FLAG antibody and 30 µl of protein-A sepharose slurry (sigma, P3391). After at least 3 hours of incubation at 4°C with agitation, the immunoprecipitants were washed three times with lysis buffer and once with 20 mM Tris, pH 8.0 and 100 mM NaCl. 35 µl of 2× SDS sample buffer were added to each immunoprecipitant and the samples were subjected to SDS-PAGE and immunoblotting analysis. For native immunoprecipitation with anti-TRAPPC9 antibody, lysates generated from approximately 2×10^7^ HEK293 cells were subjected to immunoprecipitation using antibody against TRAPPC9, or against LAMP2 (negative control). The rest of the procedures are identical to description mentioned above.

### Fluorescence microscopy

Cells grown on 12-mm coverslips were fixed in PBS with 3.7% formaldehyde for 15 min, quenched with 1% BSA in PBS, permeabilized with 0.1% Triton X-100 in PBS and blocked in PBS plus 1% BSA for the staining using anti-TRAPPC9 antibody. Others were fixed in cold methanol for 5 min and re-hydrated with 1%BSA in PBS for 20 min. The samples were incubated with primary antibodies at room temperature for 30 min, washed with 1% BSA in PBS three times, incubated with secondary antibodies for 30 min and then mounted on glass slide in Fluoromount-G (Southern BioTech, USA, 0100-01). Specific Alexa Fluor conjugated goat anti-mouse or goat anti-rabbit secondary antibodies are described in the figure legends. For colocalization of p150^Glued^ and GFP-Sec24C, after secondary antibody incubation cells needed second fixation with 3.7% formaldehyde and staining with antibody against c-Myc conjugated rhodamine [c-Myc (9E10) TRITC]. Image of stained cells were acquired with Carl Zeiss LSM-5 or Olympus FV1000 laser scanning confocal microscope equipped with 63× objective lens. The images were processed with Adobe Photoshop 7.0.

For immunofluorescence colocalization studies shown in Figure 7, the transfected COS, Hela or HEK293 cells were treated with 10 µg/ml Nocodazole for 60 minutes before staining. The degree of colocalization was calculated as a percentage of COPII fluorescent dots (presented by endogenous Sec23A or transfected GFP-Sec24C) that were also overlapped with p150^Glued^ fluorescence dots. Substantial amount of p150^Glued^ signal is diffused and cytosolic, and COPII dots overlapping with the diffused p150^Glued^ signals were excluded from our counting. Statistics analysis was done by student's two-sample T-test.

## Supporting Information

Figure S1 **Depletion of p150^Glued^ disrupts the integrity of the ER exit sites.** (A). HEK293 cells were depleted with p150^Glued^ by siRNA. The extent of depletion was monitored by immunoblotting. (B). ER exit sites (Sec31A), trans-Golgi (Golgin-97) and ERGIC & cis-Golgi (COPI) were dispersed in p150^Glued^ depleted cells. siRNA specific to luciferase was used as control depletion. Scale bar = 20 µm.
(TIF)

Figure S2 **Overexpression of GFP-tagged TRAPP subunits disrupts the star-like astral microtubule architecture in COS cells.** In each data points, at least 100 transfected cells were counted.
(TIF)

Figure S3 **TRAPPC9 partially colocalizes with p150^Glued^.** CHO cells were stained with TRAPPC9 (red) and p150^Glued^ (green). (A). p150^Glued^ signal is present in cytosol but the typical p150^Glued^ pattern of short stretch of fiber near the cell periphery is not as obvious in formaldehyde-fixed cells (see Materials and Methods). The p150^Glued^ signal in the MTOC is masked by the nearby and equally intense signal that overlaps with TRAPPC9. A significant portion of the p150^Glued^ signal is present in the cis-Golgi. (B). In nocodazole-treated cells, both TRAPPC9 and p150^Glued^ signals were dispersed into small puncta. (C) Magnified images of the puncta show close association between TRAPPC9 and p150^Glued^ (arrows). Scale bar = 20 µm (A and B);  = 5 µm (C).
(TIF)

Figure S4 **Overexpression of Sar1[H79G] clusters p150^Glued^ signals at the MTOC but disperses the Golgi-localized signals of TRAPPC9.** (A). CHO cells transfected with DNA plasmids for Myc-Sar1[H79G] and GFP-Sec24C at molar ratio 5∶1. At this ratio, essentially all the cells expressing GFP-Sec24C were also positive for Sar1[H79G] expression. The cells were stained with p150^Glued^ (left panels) or TRAPPC9 (right panels). The clustering of ER exit sites at the MTOC due to the effect of Sar1[H79G] is determined by the fluorescence pattern of GFP-Sec24C (bottom panels). Cells expressing GFP-Sec24C, and hence, Sar1[H79G], are marked with red asterisks in the top panels. (B). CHO cells were transfected with wildtype Sar1 instead. Other conditions are identical to those described in (A). Scale bar = 20 µm.
(TIF)
