# Supplementary figures and images for: TRAPPC9 Mediates the Interaction between p150Glued and COPII Vesicles at the Target Membrane
Source: PLoS One. 2012 Jan 18;7(1):e29995. doi: 10.1371/journal.pone.0029995 (PMC3261171; doi:10.1371/journal.pone.0029995)

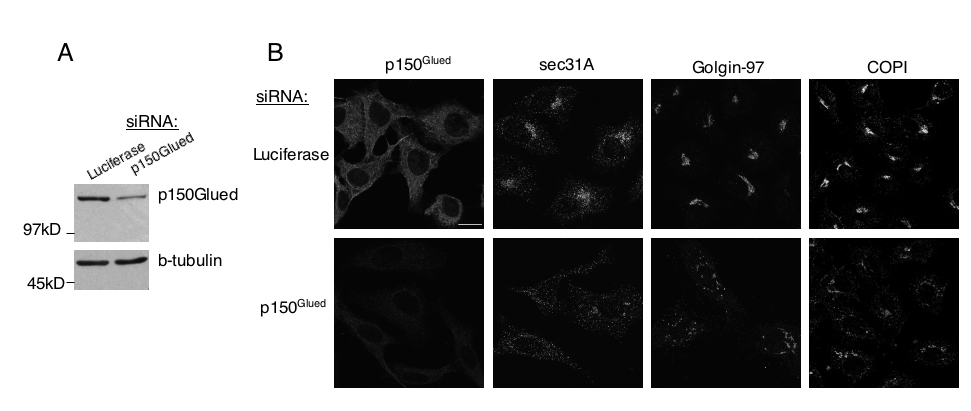

Supplement: Figure S1 — Depletion of p150Glued disrupts the integrity of the ER exit sites. (A). HEK293 cells were depleted with p150Glued by siRNA. The extent of depletion was monitored by immunoblotting. (B). ER exit sites (Sec31A), trans-Golgi (Golgin-97) and ERGIC & cis-Golgi (COPI) were dispersed in p150Glued depleted cells. siRNA specific to luciferase was used as control depletion. Scale bar = 20 µm. (TIF) [file pone.0029995.s001.tif]

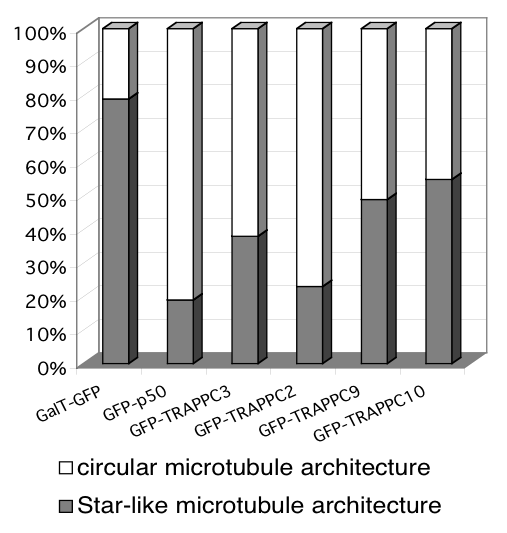

Supplement: Figure S2 — Overexpression of GFP-tagged TRAPP subunits disrupts the star-like astral microtubule architecture in COS cells. In each data points, at least 100 transfected cells were counted. (TIF) [file pone.0029995.s002.tif]

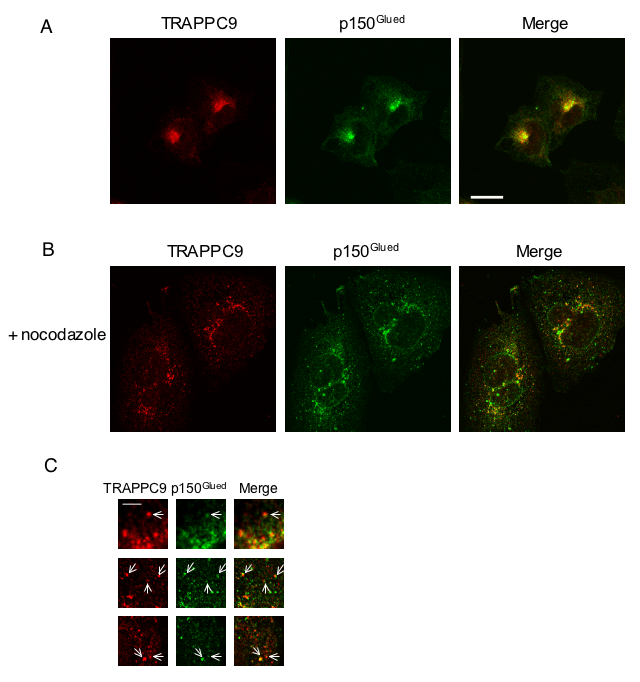

Supplement: Figure S3 — TRAPPC9 partially colocalizes with p150Glued. CHO cells were stained with TRAPPC9 (red) and p150Glued (green). (A). p150Glued signal is present in cytosol but the typical p150Glued pattern of short stretch of fiber near the cell periphery is not as obvious in formaldehyde-fixed cells (see Materials and Methods). The p150Glued signal in the MTOC is masked by the nearby and equally intense signal that overlaps with TRAPPC9. A significant portion of the p150Glued signal is present in the cis-Golgi. (B). In nocodazole-treated cells, both TRAPPC9 and p150Glued signals were dispersed into small puncta. (C) Magnified images of the puncta show close association between TRAPPC9 and p150Glued (arrows). Scale bar = 20 µm (A and B); = 5 µm (C). (TIF) [file pone.0029995.s003.tif]

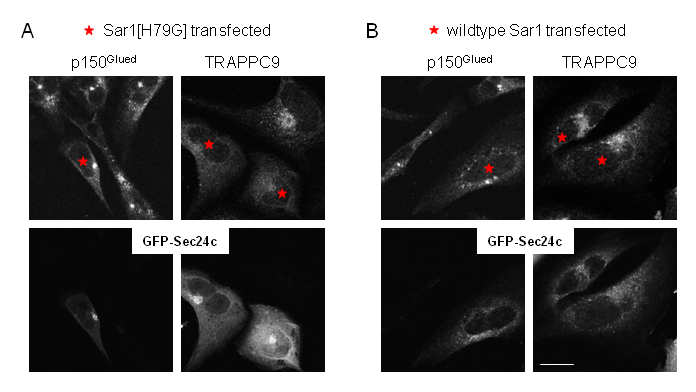

Supplement: Figure S4 — Overexpression of Sar1[H79G] clusters p150Glued signals at the MTOC but disperses the Golgi-localized signals of TRAPPC9. (A). CHO cells transfected with DNA plasmids for Myc-Sar1[H79G] and GFP-Sec24C at molar ratio 5∶1. At this ratio, essentially all the cells expressing GFP-Sec24C were also positive for Sar1[H79G] expression. The cells were stained with p150Glued (left panels) or TRAPPC9 (right panels). The clustering of ER exit sites at the MTOC due to the effect of Sar1[H79G] is determined by the fluorescence pattern of GFP-Sec24C (bottom panels). Cells expressing GFP-Sec24C, and hence, Sar1[H79G], are marked with red asterisks in the top panels. (B). CHO cells were transfected with wildtype Sar1 instead. Other conditions are identical to those described in (A). Scale bar = 20 µm. (TIF) [file pone.0029995.s004.tif]
